# Expressing exogenous functional odorant receptors in cultured olfactory sensory neurons

**DOI:** 10.1186/1749-8104-3-22

**Published:** 2008-09-11

**Authors:** Huaiyang Chen, Sepehr Dadsetan, Alla F Fomina, Qizhi Gong

**Affiliations:** 1 Department of Cell Biology and Human Anatomy, School of Medicine, University of California, Davis, California 95616, USA; 2 Department of Physiology and Membrane Biology, School of Medicine, University of California, Davis, California 95616, USA

## Abstract

**Background:**

Olfactory discrimination depends on the large numbers of odorant receptor genes and differential ligand-receptor signaling among neurons expressing different receptors. In this study, we describe an *in vitro *system that enables the expression of exogenous odorant receptors in cultured olfactory sensory neurons. Olfactory sensory neurons in the culture express characteristic signaling molecules and, therefore, provide a system to study receptor function within its intrinsic cellular environment.

**Results:**

We demonstrate that cultured olfactory sensory neurons express endogenous odorant receptors. Lentiviral vector-mediated gene transfer enables successful ectopic expression of odorant receptors. We show that the ectopically expressed mouse I7 is functional in the cultured olfactory sensory neurons. When two different odorant receptors are ectopically expressed simultaneously, both receptor proteins co-localized in the same olfactory sensory neurons up to 10 days *in vitro*.

**Conclusion:**

This culture technique provided an efficient method to culture olfactory sensory neurons whose morphology, molecular characteristics and maturation progression resembled those observed *in vivo*. Using this system, regulation of odorant receptor expression and its ligand specificity can be studied in its intrinsic cellular environment.

## Background

It is established that most of the olfactory sensory neurons (OSNs) express only one type of odorant receptor (OR) among a thousand gene choices [1,2]. Each OSN functions as a specialized sensor for the detection of diverse odorants in the environment. OSNs expressing the same OR converge their axons to the same glomerular pair in the olfactory bulb [3,4]. Thus, the one receptor-one neuron rule is essential for establishing the discrete sensory map of olfactory neuronal connections.

The ability to discriminate odorants in the environment depends on OR-ligand interactions and signaling capacity within the OSNs. Several experimental approaches for investigating OR functions have been reported [5-8]. Though heterologous systems allow rapid screening of ligand binding specificity of the ORs, validation of these results in the OSNs has been challenging without an efficient OSN culture system [9]. One of the other challenges in olfaction is to understand the regulatory mechanism for OR selection in the OSNs. Transcriptional regulatory elements that control OR expression have been identified [10,11]. In addition, a negative feedback model for achieving single OR expression in each OSN has been proposed [11-13]. The feedback inhibition of OR expression appears to require OR protein. Recent evidence indicates that the OR coding sequence plays a critical role in the suppression of multiple OR expression. Not only can endogenous OR expression be suppressed, but ectopic OR expression appears to be suppressed as well via the OR coding sequence [14].

An *in vitro *system that allows genetic manipulation will be helpful in determining the genes and the functions of their products that are involved in the regulation of OR expression and function. Several primary OSN culture techniques have been reported [15-17]. Studies using cultured OSNs have primarily focused on investigating the regulatory mechanism of neurogenesis and differentiation, though responses to odorants and signaling molecule functions were reported in primary OSN cultures [18,19]. However, the expression of ORs and the genetic manipulation of OR expression in cultured OSNs have not been reported to date. Here we describe an OSN culture system that allows gene expression manipulation, monitoring of OR expression and testing of OR-ligand specificity. Lentiviral vectors are used to achieve a high percentage of infection of cultured OSNs and to express ectopically genes of interest. Using this system, we observed that OSNs allow expression of two ORs when introduced by lentiviral-mediated gene transfer.

## Results

### Primary olfactory sensory neurons express olfactory specific markers *in vitro*

**Figure 1:**
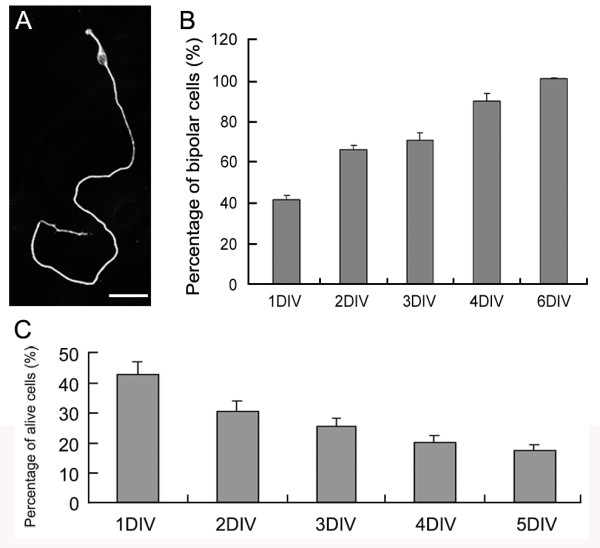
**Primary olfactory sensory neuron culture**. A) Olfactory sensory neurons were identified by β-tubulin III expression and were bipolar with a short dendrite-like and a long axon-like process, resembling their *in vivo *morphology. B) Percentage of bipolar neurons among all neuronal cells was at 41.1% after one day *in vitro *(DIV). The percentage of bipolar neurons increased to 99.7% at 6DIV. C) The average survival rate of the cultured olfactory sensory neurons, when compared to plating cell density, was 41.6 ± 4.2% at 1DIV, 29.9 ± 3.1% at 2DIV, 24.7 ± 2.7% at 3DIV, 19.3 ± 2.1% at 4DIV and 16.9 ± 1.8% at 5DIV. Values are mean ± standard deviation from three independent experiments at each data point. Bar = 30 μm.

To establish an *in vitro *system to investigate the regulatory mechanisms of odorant receptor expression and function, we first extensively validated our primary OSN culture system. In our cultures, OSNs isolated from olfactory neuroepithelia (OE) of embryonic or neonatal mice were maintained on a confluent layer of cortical astrocytes. The neuronal identity of the cultured cells was characterized by their expression of neuronal specific β-tubulin III. Neurons in the cultures exhibited characteristic bipolar morphology with a short dendrite-like process and a long and thin axon-like neurite (Figure 1A). Neurons with multipolar morphology were initially present in the cultures, but gradually disappeared. At 1 day *in vitro *(1 DIV), 41.1% of the neurons were bipolar. The percentage of bipolar neurons increased to 65.4% at 2 DIV, 70.0% at 3 DIV, 88.9% at 4 DIV and 99.7% at 6 DIV (Figure 1B). While the percentage of the bipolar neurons increased, the density of the neurons in the cultures, which were plated at 1.5 × 10^5 ^cells/cm^2 ^initially, decreased from 41.6% of the plating density at 1 DIV to 16.9% at 5 DIV (Figure 1C). These data suggest that the bipolar neurons survived better than other types of neurons in our system.

**Figure 2:**
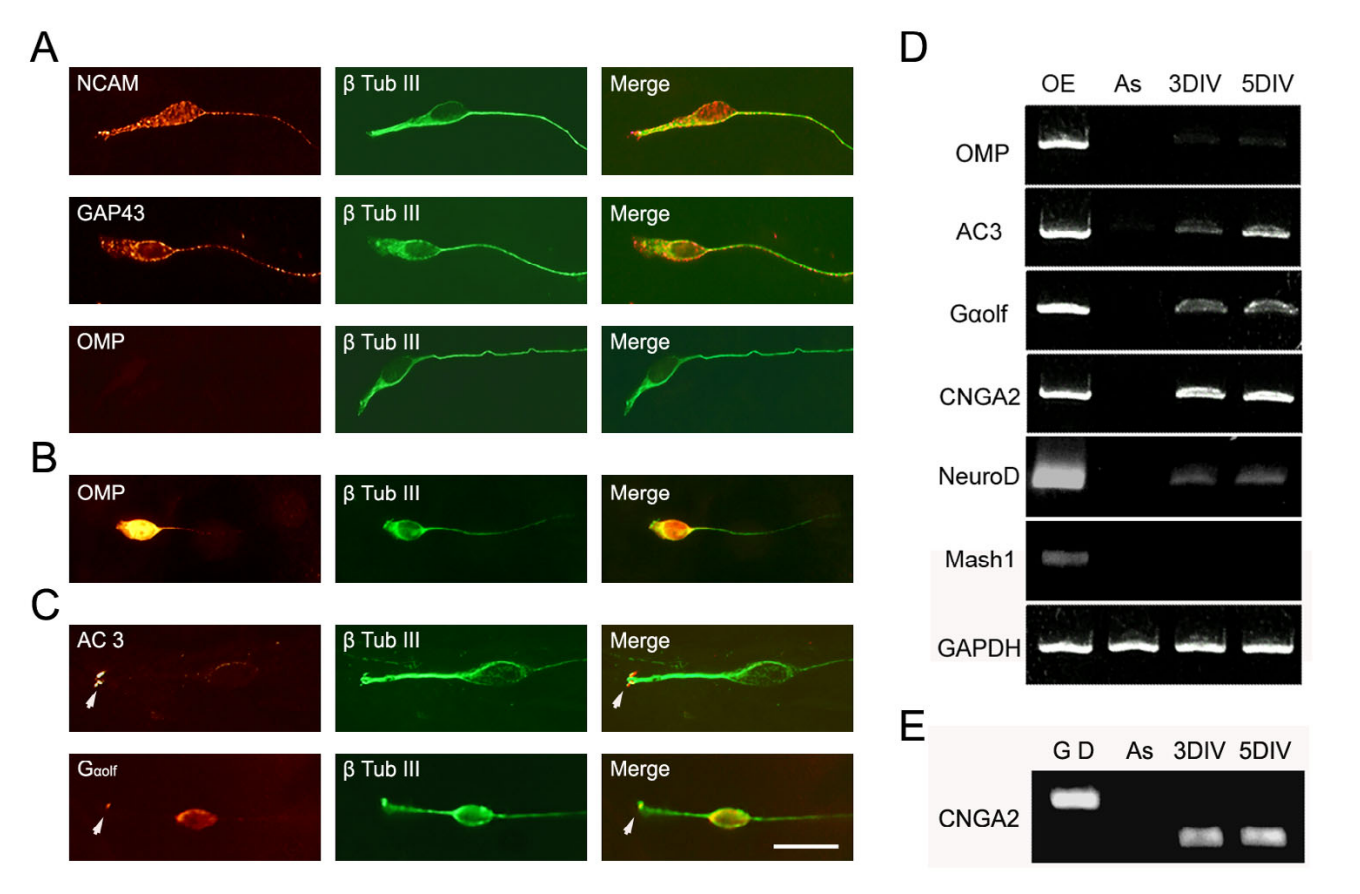
**Cultured olfactory sensory neurons express characteristic markers**. A) Cultured olfactory sensory neurons identified by β-tubulin III (β-Tub III) expression (green) also express neuronal cell adhesion molecule (NCAM; red) and GAP-43 (red) and olfactory marker protein (OMP; red) at 3 DIV. B) OMP positive cells were identified at 8 days *in vitro *(DIV). C) Adenylyl cyclase (AC)3 (arrowhead) and G_αolf _(arrowhead and cell body) expression (red) at 5 DIV. D) OMP, AC3, G_αolf_, cyclic nucleotide gated channel Ay subunit (CNGA2) and NeuroD expression were detected by RT-PCR at 3 DIV and 5 DIV. Mash1 expression was not detected at 3 DIV or 5 DIV. GAPDH expression served as quantity control. Olfactory epithelium (OE) cDNA was used as a positive control and feeder layer astrocyte (As) cDNA was used as a negative control. E) CNGA2 primers were designed to span an intron and, therefore, were able to discriminate PCR products amplified from genomic DNA (G D), which is 710 bp, or olfactory sensory neuron culture cDNA, which is 429 bp. Bar = 20 μm.

The initial decrease in neuronal numbers in the cultures may reflect lack of survival of the mature OSNs dissociated from OE. Olfactory marker protein (OMP) expression is the hallmark for mature olfactory sensory neurons. To evaluate the survival of OMP positive cells, we immunostained the OSN cultures from postnatal day (P)0 OE at different time points and counted the numbers of OMP positive cells. OMP positive neurons were initially present 1 hour after plating. Within eight randomly chosen fields (area = 0.18 mm^2^), 40 OMP positive cells were observed among 157 total neurons, which is around 25.5% at 1 hour after plating. At 1 DIV, the number of OMP positive cells was dramatically decreased in the cultures. Among 26,000 β-tubulin III positive neurons, only 10 OMP positive cells were observed. At 3 DIV and 5 DIV, no OMP cells were seen in the cultures (Figure 2A). We also noticed that OMP signals were barely detectable by RT-PCR at 3 DIV and 5 DIV (Figure 2D). Though few in number, OMP positive neurons reappear at 8 DIV (Figure 2B). These data suggest that mature OMP positive OSNs dissociated from OE lack the ability to survive under our culture conditions, but immature OSNs in the cultures have the potential to differentiate into OMP positive neurons when cultured for 8 DIV or longer.

Do the bipolar neurons display molecular markers of OSNs? We observed that all neurons in the cultures, identified by β-tubulin III expression, expressed neuronal cell adhesion molecule (NCAM; Figure 2A), which is expressed by OSNs *in vivo *[17]. In addition, at 3 DIV, all cultured neurons were also positive for GAP-43 immunostaining, which is a marker for immature OSNs (Figure 2A). Adenylyl cyclase (AC)3 and G_αolf _are key players in mediating olfactory signals and are expressed in OSN dendrites and axons during development [20]. Both AC3 and G_αolf _expression were detected in cultured OSNs at 3 DIV by both immunostaining and RT-PCR (Figure 2). AC3 and G_αolf _protein were distributed throughout the OSNs, including the cell body, and dendrite- and axon-like processes at 3 DIV. At 5 DIV, however, AC3 was primarily concentrated at the tip of the short dendritic like processes, and at low levels in the cell body and their axons. At 5 DIV, G_αolf _immunostaining signal was detected in the cell body and concentrated at the tip of the short dendritic-like processes as well (Figure 2C). We observed that AC3 and G_αolf _transcript levels were higher at 5 DIV when compared to those at 3 DIV (Figure 2D). Cyclic nucleotide gated channel A2 subunit (CNGA2) was also expressed in the cultured OSNs at 3 DIV and 5 DIV (Figure 2D). Expression of progenitor and neuronal precursor markers were also examined using RT-PCR. Both Mash1, an amplifying progenitor marker, and Ngn1, a neuronal precursor marker, are expressed in OE *in vivo *[21]. However, we did not detect expression of these two genes in the OSN cultures at 3 DIV and 5 DIV. NeuroD, which is expressed in immature OSNs *in vivo *[22], was detected in the OSN cultures at both 3 DIV and 5 DIV (Figure 2D).

**Figure 3:**
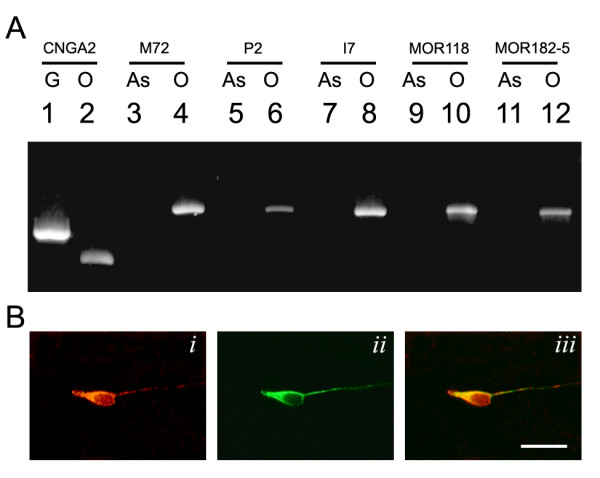
**Primary olfactory sensory neurons express endogenous odorant receptors**. A) Odorant receptor transcripts were detected in cultured olfactory sensory neurons by RT-PCR. Cyclic nucleotide gated channel Ay subunit (CNGA2) primers were designed to discriminate PCR products amplified from genomic DNA (710 bp; G) or olfactory sensory neuron culture cDNA (429 bp; O). The odorant receptor transcripts were not detected in cortical astrocytes (As). All selected odorant receptor transcripts, including M72, P2, I7, MOR118 and MOR182-5, were detected in cultured olfactory sensory neurons at 3 days *in vitro *(DIV). B) Odorant receptor protein expression was detected in cultured olfactory sensory neurons. Olfactory sensory neurons were identified by β-tubulin III expression (green in *ii*). MOR42-3 immunostaining positive neurons (red in *i*) were detected among cultured olfactory sensory neurons (merge in *iii*) at 3 DIV. Bar = 20 μm.

Do cultured OSNs express odorant receptors? In this study, we examined the expression of five different ORs, I7, P2, M72, MOR118-1 and MOR182-5. The chromosome locations and the onset of expression of each of these ORs during development are different [23,24]. In cultured OSNs, all five OR transcripts were detected by RT-PCR at 3 DIV (Figure 3). As controls, OR transcripts were not detected in the cortical astrocyte feeder layer cells. To examine whether OR proteins were produced and to determine the number of OSNs expressing a selected OR in the cultures, we performed immunostaining using an OR-specific antibody. Polyclonal MOR42-3 antibody was used to detect expression of this OR in the OSN cultures. The MOR42-3 antibody was first validated in the OE; we observed that the MOR42-3 antibody recognized a small subset of olfactory sensory neurons in the zone 1 region of the OE, consistent with published results [1]. MOR42-3 immunoreactivity was localized in the OSN cell bodies, dendrites and ciliary processes. At 3 DIV, MOR42-3 positive neurons were observed. MOR42-3 immunoreactivity, similar to that of the OE, was distributed in the OSN cell body and concentrated in the dendrite-like process (Figure 3B). Among 4 × 10^4 ^neurons screened, MOR42-3 immunopositive neurons were found at the frequency of 0.06% (24 out of 1 × 10^4^) in the OSN cultures.

### Efficient gene transfer using lentiviruses

**Figure 4:**
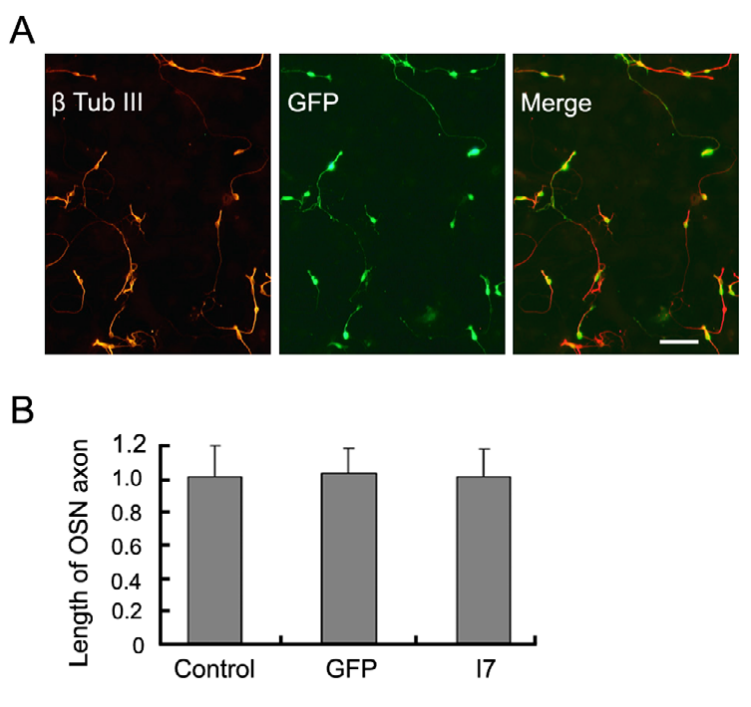
**Lentiviral vector mediated gene transfer in primary olfactory sensory neurons**. A) When 10^5^pfu/ml recombinant enhanced green fluorescent protein (EGFP)-expressing lentiviral vectors were added to the primary olfactory sensory neuron culture, all β-tubulin III (β-Tub III) positive neurons (red) expressed EGFP (green). B) Lentiviral infection and ectopic expression of EGFP do not alter olfactory sensory neuron (OSN) morphology or axon length. When normalized as 1 ± 0.19 in the control (n = 74), OSN axon average lengths were 1.02 ± 0.15 (n = 56; *t*-test *p *> 0.8) in GFP lentiviral vector-infected cultures and 1.00 ± 0.17 (n = 60; *t*-test *p *> 0.9) in mouse I7 lentiviral vector-infected cultures. Values are mean ± standard error from three independent experiments. Bar = 30 μm.

The difficulty of culturing and genetically manipulating cultured OSNs has impeded the progress of using *in vitro *approaches to investigate molecular mechanisms of OSN differentiation and odorant signaling. Though various transfection techniques and a biolistic gene transfer technique for cultured OSNs have been attempted, the efficiency of the gene transfer and survival rate of the OSNs were low (unpublished data; see also [25]). In this study, we evaluated the effectiveness of lentiviral vector-mediated transfection in OSN cultures. VSV-G pseudotyped lentiviruses carrying an enhanced green fluorescent protein (EGFP) expression cassette were added to the OSN cultures at the time of the plating. When 10^5^pfu/ml of lentiviruses were added to the cultures, we observed that nearly 100% of β-tubulin III positive OSNs expressed GFP at 3 DIV (Figure 4A). The morphology of the OSNs infected by lentiviruses was not changed, at this defined viral concentration, compared to that of non-infected cultures (Figure 4A). The axon lengths of the lentiviral infected OSNs were not significantly different compared to those of control non-infected cultures; when axon lengths of the control OSNs were normalized to 1 (standard error (SE) = 0.18), the average axon length of GFP lentiviral vector-infected neurons was 1.02 ± 0.15 (*t*-test, *p *> 0.8) (Figure 4B). Therefore, lentiviral infection allows high efficiency of gene transfer and does not alter survival and morphology of the OSNs in culture.

### Functional expression of I7 in cultured OSNs

**Figure 5:**
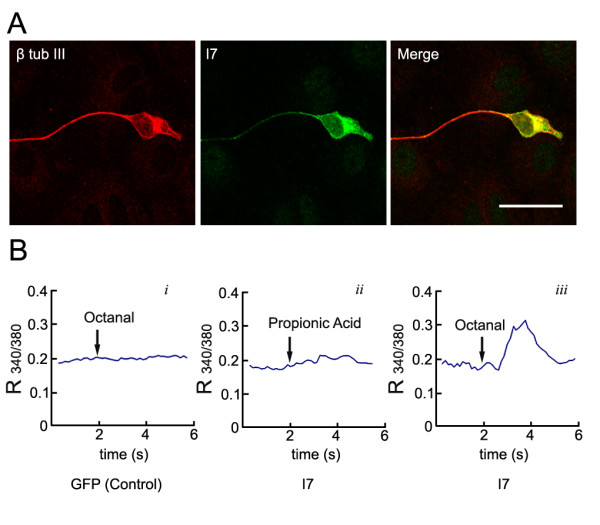
**Over-expressed mouse I7 odorant receptors are functional**. A) A mouse I7-green fluorescent protein (GFP) fusion protein expression cassette was introduced into cultured olfactory sensory neurons by recombinant lentiviral vector. I7-GFP expression recognized by GFP immunostaining (green) was detected in the olfactory sensory neurons identified by β-tubulin III expression (red). I7-GFP is distributed in the olfactory sensory neuron cell body, axon- and dendrite-like processes. B) Time course of the ratio of fura-2 fluorescence intensity at 340 nm and 380 nm (R_340/380_) recorded from olfactory sensory neurons expressing GFP alone (B*i*) or I7-GFP (B*ii and *B*iii*) in the presence of 100 μM octanal (B*i *and B*iii*) or 100 μM propionic acid (B*ii*). Arrows indicate timing of application of octanal or propionic acid. Each trace represents an average of R_340/380 _time courses recorded simultaneously from five to seven cells in a representative experiment. Bar = 20 μm.

With the efficient lentiviral vector-mediated gene transfer technique, we then asked whether we could express functional OR in cultured OSNs. Expression of functional OR in heterologous cells has proven to be challenging due to their inefficient trafficking to the cell surface [5,26]. The expression of exogenous ORs in OSNs in the nasal epithelium using adenoviral vector has been reported and the rat odorant receptor I7 introduced to OSNs *in vivo *has proven to be functional [9]. Adenoviral vectors, however, showed toxicity towards the cultured OSNs and significantly decreased OSN survival at 3 DIV. Recombinant lentiviruses carrying the mouse I7 coding sequence fused with EGFP were added to the culture at the concentration of 10^5^pfu/ml. All OSNs in the lentiviral-infected cultures expressed I7-GFP detected by GFP immunostaining. I7-GFP was distributed in the OSNs, including the cell body, and the axon- and dendrite-like processes (Figure 5A).

To examine whether ectopically expressed I7 in cultured OSNs are functional receptors, we tested whether these infected OSNs would respond to octanal (octyl aldehyde), which is one of the documented I7 odorant ligands. It is reported that functional odorant receptors can trigger an increase in [Ca^2+^]_i _in OSNs when stimulated by their odorant ligands. To measure [Ca^2+^]_i_, cultured OSNs were loaded with fura-2/AM and cells with stable basal levels of [Ca^2+^]_i _were selected for recording. As a control, we first examined whether cultured OSNs infected with recombinant GFP-expressing lentivirus respond to the presence of octanal. Octanal at 100 μM rarely elicits responses in a majority of the cultured OSNs expressing GFP alone (Figure 5Bi). Among 64 cells tested, one cell showed a [Ca^2+^]_i _increase in response to octanal, suggesting that this cell may express endogenous functional receptors. It has been shown that propionic acid is not a ligand for the I7 receptor [9]. In OSN cultures infected with I7 lentiviral vectors, none of the I7-expressing cells showed responses to 100 μM propionic acid (n = 28; Figure 5Bii); however, all I7-expressing cells tested displayed an increase in [Ca^2+^]_i _in response to 100 μM octanal (n = 31; Figure 5Biii). This result indicates that ectopically expressed mouse I7 in cultured OSNs are functional ORs.

### Expressing functional I7 does not alter OSN axon extension

ORs play important roles in OSN axon convergence. The site of the axon convergence could be influenced by the level of OR expression suggested by OR knock-in animal models. Expression of the OR has also been shown to be linked to the expression of certain cell surface adhesion molecules [27]. In our culture system, OSN axons migrate on the surface of the feeder layer astrocytes. Whether over-expressing ORs influences the rate of OSN axon extension has not been examined. Utilizing the OSN cultures, we examined this question by comparing the axon length of the OSNs between I7-expressing and control cultures. At 3 DIV, when the average axon length in the control noninfected cultures was normalized to 1, the average axon length of the I7 lentiviral vector-infected culture was 1.00 ± 0.17 (mean ± SE, n = 56, *t*-test *p *> 0.9; Figure 4B). Therefore, over-expressing I7 does not alter the extension of the OSN axons. In addition, when OSNs are grown at a density of the 5 × 10^4^/cm^2^, we did not observe any fasciculation between I7-expressing OSN axons.

### Expression of two exogenous ORs is possible in cultured OSNs

**Figure 6:**
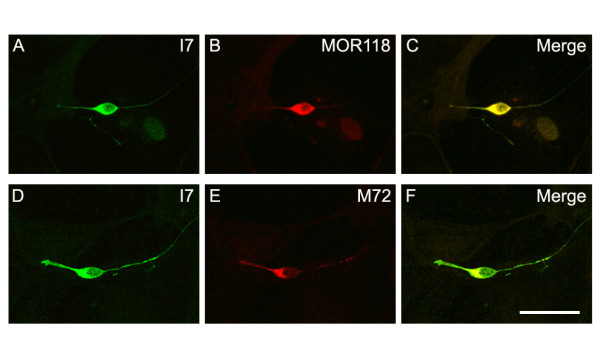
**Expression of two exogenous odorant receptors in cultured olfactory sensory neurons**. olfactory sensory neurons (OSNs) were infected with I7-green fluorescent protein (GFP) and MOR118-mCherry or I7-GFP and M72-mCherry recombinant lentiviral vectors. The expression of the odorant receptors were detected by GFP or RFP immunostainings (A-F). A, B) At 7 days *in vitro *(DIV), all OSNs expressing I7 (A) also express MOR118 (B). D-F) Similarly, olfactory sensory neurons expressing I7 (D) can also express M72 (E) in the same cells (F). Bar = 30 μm.

In mammals, one OSN selectively expresses one type of OR from among approximately 1000 OR genes. We attempted to co-express two ORs using two lentiviral vectors, each expressing an OR under the control of an exogenous human ubiquitin promoter. In this set of experiments, lentiviral vectors expressing a I7-GFP fusion protein were added to the cultures simultaneously with P2-mCherry, M72-mCherry, or MOR118-mCherry expressing viruses for the convenience of visualization. We examined ectopic OR expression by GFP and mCherry immunostaining at 3 DIV, 5 DIV, 7 DIV, and 10 DIV. All expression combinations described above showed identical results. Using the previously defined viral titer, all OSNs in the culture expressed both ectopic ORs and no single ectopic OR expressing OSNs were observed at 3 DIV. The expression of the ectopic ORs did not appear to change with regard to their co-localization and levels in all the OSNs examined at 7 DIV when compared with those at 3 DIV (Figure 6). This co-expression persists till 10 DIV, the longest time investigated. No significant changes in the levels of each individual OR were observed.

## Discussion

In this study we report that cultured primary OSNs express endogenous ORs along with other characteristic odorant signaling molecules. Lentiviral vector-mediated gene transfer allows efficient and successful ectopic expression of functional ORs in culture. The mouse OSN culture system described here will enable future studies of OR ligand specificity and transcription regulation within its intrinsic cellular environment.

### *In vitro *characteristics of primary OSNs resemble their *in vivo *characteristics

In the OSN culture system reported here, several modifications were made to enhance the survivability and morphology of the OSNs compared to other published studies [15,28]. Dissociated OE cells were plated on a feeder layer of confluent astrocytes instead of directly on matrix coated coverglass to enhance their attachment, survivability and differentiation. Defined, serum-free media supported the survival of neurons and inhibited cell proliferation and neurogenesis. Consistent with previous reports, OMP expression was present immediately after the plating and was undetectable at 3 DIV [15]. A rapid decrease in OMP positive cells indicated that the culture condition does not support the survival of mature OSNs dissociated from the OE. In addition, a larger numbers of multipolar neurons were present at 1 DIV. The percentage of bipolar neurons increased almost 100% from 1 DIV to 6 DIV, while the number of multipolar neurons decreased. This change in the cell population in the OSN cultures suggests that mature OMP positive neurons and multipolar neurons were not well supported under our culture conditions. We do not have evidence at this point as to whether the multipolar neurons represent certain populations of OSNs. All multipolar neurons were GAP43 and NCAM positive in the cultures. Since all OSNs in the cultures were positive for GAP43 staining at 3 DIV, the OSNs in the cultures are most likely immature neurons. Expression of neuronal progenitor markers Mash1 and Ngn1 was not detected at 3 DIV in the culture. This observation is consistent with the idea that the culture conditions do not support neurogenesis. In addition, it also suggests that the precursors dissociated from the OE either did not survive in the cultures or progressed along their differentiation path to become immature OSNs at 3 DIV. The small number of OMP positive neurons detected at 8 DIV leads us to suggest that OMP positive mature OSNs can be generated under these defined culture conditions.

OSNs are bipolar neurons with their dendritic process extending towards the surface of the nasal cavity. At the tip of the dendrite, the ending swells into a knob-like morphology and ciliary processes extend out from the dendritic-knob. OR proteins and signaling proteins mediating the odorant activity are found to be enriched in the ciliary processes [19,29]. In cultured OSNs, signaling molecules exhibit preferential localization at the tip of the dendritic-like process. G_αolf _and particularly AC3 were both initially distributed throughout the cell body, axon and dendritic processes, and eventually accumulated at the tips of the dendrites. The concentrated subcellular localization of AC3 and G_αolf _at the dendritic tip is evident at 5 DIV, and resembles their *in vivo *distribution [30,31]. Though olfactory signaling molecules and several OSN specific markers were present, the cultured OSNs did not recapitulate all cellular and molecular characteristics of mature OSNs *in vivo*. Only a small proportion of OMP positive neurons differentiated from immature neurons and only at 8 DIV or later in the culture. It is possible that OMP expression may be partially dependent upon the presence of the cellular targets of the OSNs. Overall, this culture technique provided an efficient method to culture OSNs, whose morphology, molecular characteristics and maturation progression in culture resembled those observed *in vivo*.

### Odorant receptor expression and function in cultured OSNs

Based on the developmental timing and the distribution of OR expression in the epithelium, it was concluded that OR expression precedes OMP expression [32]. As it is not practical to examine the expression of all ORs, we selected five ORs that represent OR genes located on different chromosomes that are turned on at different developmental time points [24,23]. All ORs examined were expressed in our OSN culture. Since cultured OSNs are obtained from embryonic day (E)17-P0 mouse OE, it is possible that all ORs are expressed by P0. A recent study of the septal organ demonstrated clearly that OR expression is temporally asynchronous [33]. The onset of OR expression in the septal organ can span several days from E16 to P0. Similar expression onset asynchrony is also reported in the main OE [23]. The density and distribution of OR expressing OSNs also vary within the OE. Therefore, it is possible that not all ORs can be detected in the cultured OSNs.

Investigations of OR ligand specificity have been challenging due to the difficulty of expressing ORs in heterologous cells. Amino-terminal modification by adding a rhodopsin tag to the OR coding sequence enhanced the ability of OR to be transported to the plasma membrane [5]. However, this extracellular modification may alter ligand receptor binding specificity [34]. Accessory proteins, including odorant receptor transporting proteins and Ric8b, are known to aid in the efficiency of OR expression [8]. Combinations of these accessory factors and signal transduction molecules allow expression of a subpopulation of ORs and subsequent high throughput screening for their ligand. However, many intrinsic differences within the heterologous systems, including availability and subcellular localization of the signaling molecules and the concentration of OR expressed, could all influence the experimental outcome. ORs expressed in their intrinsic environment by OSNs presumably behave most closely to their *in vivo *state [9].

## Conclusion

In this study, we report an efficient technique for ectopic expression of ORs in OSNs. Using lentiviral vectors, we report close to 100% infection of the cultured OSNs. This high infection efficiency allows not only easy access to the OR-expressing cells but also monitoring of changes using molecular and biochemical approaches. In addition, we provide evidence that the ectopically expressed mouse I7 is functional. Though an earlier report indicated that mouse I7 is more sensitive to heptanol, later evidence argued for mouse I7 to respond to multiple aldehyde compounds [34]. To define the functionality of ectopic mouse I7, but without the intent to discriminate ligand-receptor specificity among aldehydes, we used a higher concentration of octanal (100 μM) as an agonist for receptor-induced odorant responses. Calcium imaging experiments confirmed that I7-expressing OSNs responded to odorant stimulation. Consistent with the finding that endogenous ORs are expressed by cultured OSNs, we detected occasional responsive cells among control cultures. This observation suggests that endogenous ORs are functional receptors as well. The cultured OSN system reported here provides an efficient way to express functional ORs and to study ligand specificity in their intrinsic cellular environment.

## Materials and methods

### Animals

C57/BL6 mice were purchased from Charles River Laboratories (Wilmington, MA). Embryonic day 0 (E0) was defined by the day when the copulation plug was detected in the morning. The day of birth was defined as P0. All experimental procedures were conducted according to institutional and NIH guidelines and were approved by the Institution Animal Care and Use Committee.

### Cortical astrocyte feeder layer

Primary astrocytes were isolated from P0 mouse neocortex and cultured according to a published protocol [35]. Before plating OSNs, astrocytes were plated and allowed to reach confluency on poly-D-lysine and laminin coated glass coverslips. The purity of the astrocytes was evaluated by immunostaining a sample of the astrocyte culture for glial fibrillary acidic protein to identify the astrocyte population and β-tubulin III to determine the presence of neurons. When no neurons were observed, astrocytes were used for OSN culture.

### Dissociated primary OSN culture

E17 to P0 OE were dissected from the nasal cavity. After 40 minutes' incubation in 2 mg/ml dispase at room temperature, OE were separated from the underlining stroma using fine tungsten needles. The OE were incubated in Waymouth's MB 752/1 medium with N2 supplement (Invitrogen, Carlsbad, CA, USA) at 37°C for 2 hours before treatment with 0.05% trypsin for dissociation. After trypsinization, dissociated cells were plated at a density of 5 × 10^4 ^cells/cm^2 ^on the confluent astrocyte feeder layer. Dissociated OSNs were maintained in Waymouth's MB 752/1 medium with N2 supplement for up to 10 days.

### Production of lentiviral vectors

The lentivirus backbone (pFUW) was kindly provided by C Lois [36]. To generate pFUW-EGFP, we first modified pEGFP-N1 (BD Biosciences, San Jose, CA, USA) by eliminating two restriction sites, *Hpa*I and *Mfe*I, via site-directed mutagenesis using primers GFP-MH2 and GFP-MH2R (Table 1). EGFP sequences were then amplified from the modified pEGFP-N1 by PCR using primers GFP-LTF and GFP-LTR (Table 1) and cloned into the *Eco*RI site of the pFUW to obtain pFUW-EGFP. The I7 open reading frame sequence was obtain by PCR from mouse OE cDNA using primers I7-F and I7-R (Table 1) and cloned into pCR-blunt TOPO-II (Invitrogen) to generate pCR-I7. After the sequence was confirmed, the mouse I7 open reading frame was cloned into pFUW-EGFP between the *Hpa*I and *Eco*RI sites to produce pFUW-I7-EGFP. To generate pFUW-mCherry, primers GFP-LTF and mCRY-R were used to amplify the mCherry sequence from pRSET-B mCherry [37] and cloned into pFUW. Similarly, pFUW-P2-mCherry, pFUW-MOR118-mCherry and pFUW-M72-mCherry were generated as described above using gene-specific primers (Table 1). Recombinant lentiviruses were produced as described [36]. Titers of the viruses were between 10^8 ^and 10^10^pfu/ml. When infecting OSNs in culture, 10^5^pfu/ml viruses were added at the time of plating in all experiments. While growing astrocytes in the non-confluent cultures were capable of being infected by lentiviruses, similar to the dissociated OSNs, astrocytes in the confluent feeder layers were rarely infected. This characteristic phenomenon allows the investigation of lentiviral-mediated expression in the OSNs in our culture system.

**Table 1:** Primer sequences for this study

Primer name	Sequence
Eco OMP	GAATTCATGGCAGAGGACGGGCCACAGAAG
OMP Xho	CTCGAGTCAGAGCTGGTTAAACACCACAG
AC3F	CAGCCAGCAGCCAACATGCCG
AC3R	CTTCCAGCTCATCCTGCTGCTG
GolfF2	ATGGGCCTATGCTACAGCCTGCGG
GolfR2	CTGACCGGAACTGGTTCTCAGGGTTG
CNGF2	GGTGCTGGATTACTTCTCAGACAC
CNGR	CCAGGTAGCCATATTCAGGGTCAG
GAPDH-F	ATGGTGAAGGTCGGTGTGAACG
GAPDH-R	AGTGATGGCATGGACTGTGGTC
M72-F	GTTAACATGGCTGCAGAGAATCAATC
M72-R	GAATTCAAAGACTCTTCTCCTCAGTG
P2-F	GGATCCATGACCTGGGGAAACTGGAC
P2-R	GAATTCTAGTTTCTGAGGGCCCAGAG
I7-F	GTTAACATGGAGCGAAGGAACCACAC
I7-R	GAATTCACCATCTCTGCTGGATTTC
OR118F	ATGGCGAACAGCACTACTGTTACTG
OR118R	TGTCTGGCTGAACTTTTGGAACTTGC
OR203F	ATGGAGGTGAACAGGACCCTGGTGACT
OR203R	TGGCTTCATGATTTTTCTCAGAGCC
GFP-LTF	GAATTCGCCACCATGGTGAGCAAGGGCG
GFP-LTR	CAATTGCTTAAGATACATTGATGAGTTTGG
GFP-MH2	AATGAATGCAATAGTTGTTGATAACTTGTTTATTG
GFP-MH2R	CAATAAACAAGTTATCAACAACTATTGCATTCATT
mCRY-R	GCGGCCGCTTTACTTGTACAGCTCGTCCATGCC
NeuroD-F	CAAGGTGGTACCTTGCTACTCCAAG
NeuroD-R	GGAATAGTGAAACTGACGTGCCT
Mash-F1	TGCCAGGCTCTCCTGGGAATGG
Mash-R1	CTGGTTCGGATAGATACAAATAG

In the RT-PCR experiments, olfactory marker protein transcripts were amplified by primers Eco OMP and OMP Xho, Adenylyl cyclase 3 by AC3F and AC3R, G_αolf _by GolfF2 and GolfR2, cyclic nucleotide gated channel A2 subunit by CNGF2 and CNGR, NeuroD by NeuroD-F and NeuroD-R, Mash1 by Mash-F1 and Mash-R1, GAPDH by GAPDH-F and GAPDH-R, receptor M72 by M72-F and M72-R, I7 receptor by I7-F and I7-R, P2 receptor by P2-F and P2-R, MOR118 by OR118F and OR118R, and MOR182-5 by OR203F and OR203R.

### Immunohistochemistry

Cultured cells were fixed with 4% paraformaldehyde for 15 minutes and immunostained as described previously [38]. The sources and the dilutions of the primary antibodies were: monoclonal mouse anti-β-tubulin III 1:300 (Sigma, Saint Louis, MO, USA); rabbit anti-GAP-43 1:200 (Chemicon, Temecula, CA, USA); mouse anti-NCAM 1:100 (Developmental Studies Hybridoma Bank, Iowa City, IA, USA); chicken anti-OMP 1:1000 [39]; rabbit anti-AC3 1:100 (Santa Cruz Biotechnology, Santa Cruz, CA, USA); rabbit anti-Gαolf 1:100 (Santa Cruz Biotechnology); rabbit anti-GFP 1:3000 (Invitrogen); rabbit anti-RFP 1:250 (MBL International Corp., Woburn, MA, USA); rabbit anti-MOR42-3 1:2000 (Osenses Pty Ltd, Flagstaff Hill, SA, Australia)

### RT-PCR analysis

Total RNAs were extracted from the OSN cultures, including astrocyte feeder layer cells and astrocyte feeder layer only cultures, using TRIZOL reagent (Invitrogen). Template cDNAs were obtained by reverse transcription (RT) using oligo dT primers. Detection of cDNAs was done by PCR reactions using primers listed in Table 1. All RT-PCR products were confirmed by sequence analysis.

### Single-cell intracellular calcium imaging

Before each experiment, cultured OSNs were loaded with 1 μM fura-2/AM (Molecular Probes/Invitrogen, Eugene, OR, USA) for 5 minutes in Ringer's solution containing 0.02% (v/v) pluronic acid (Molecular Probes/Invitrogen). After washing, cells were then incubated for an additional 30 minutes at 37°C. Coverslips with fura-2- loaded OSNs were then placed into the recording chamber continuously perfused with Ringer's solution and mounted on a Zeiss Axiovert 200 inverted microscope (Zeiss, Thornwood, NY, USA). Solution exchange was accomplished via a gravity-driven perfusion system, which allowed complete solution exchange in the recording area within 2 to 5 seconds. Fluorescence images of fura-2- loaded OSNs were acquired using a SenSys CCD camera (Roper Scientific, Tucson, AZ, USA). A lambda DG-4 filter changer (Sutter Instrument, Novato, CA, USA) was used for switching between 340 nm and 380 nm excitation wavelengths. Changes in intracellular calcium concentrations ([Ca^2+^]_i_) were monitored as changes of the ratio of the fura-2- fluorescence intensity recorded at 340 nm and 380 nm excitation wavelengths (R_340/360_). All experiments were performed at room temperature. Data acquisition and analysis were performed using MetaFluor v7.0 software (Universal Imaging, Downingtown, PA, USA). In each experiment transfected cells were identified by GFP fluorescence and time courses of R_340/360 _were simultaneously recorded from five to seven transfected cells.

## Abbreviations

AC: adenylyl cyclase; CNGA2: cyclic nucleotide gated channel A2 subunit; DIV: days *in vitro*; E: embryonic day; EGFP: enhanced green fluorescent protein; NCAM: neuronal cell adhesion molecule; OE: olfactory neuroepithelia; OMP: olfactory marker protein; OR: odorant receptor; OSN: olfactory sensory neuron; P: postnatal day; SE: standard error

## Competing interests

The authors declare that they have no competing interests.

## Authors' contributions

HC carried out all the experiments in this study, and participated in acquisition, analysis and interpretation of the data. SD and AF participated in the design and execution of the calcium imaging study. AF participated in drafting the manuscript. QG conceived of the study, and participated in its design, coordination, interpretation of the data and drafting of the manuscript. All authors read and approve the final manuscript.
